# Assessment of health management strategies and socioeconomic factors contributing to obesity in urban populations

**DOI:** 10.6026/973206300215038

**Published:** 2025-12-15

**Authors:** Balvinder Singh, Vijayalakshmi Eruva, Amrit Podder

**Affiliations:** 1 Department of Physiology, Santosh Medical College and Hospital, Ghaziabad, Uttar Pradesh, India; 2 Department of Community Medicine, RIMS Adilabad, Adilabad, Telangana, India; 3 Department of Physiology, Teerthanker Mahaveer Medical College & Research Centre, Teerthanker Mahaveer University, Moradabad, Uttar Pradesh, India

**Keywords:** Healthcare access, income level, obesity, physical activity, socioeconomic factors

## Abstract

Obesity in urban populations is a growing public health concern, influenced by various health management strategies and socioeconomic factors. Therefore, it is of interest to assess the health management strategies and socioeconomic factors contributing to obesity in urban populations. Data were collected from 150 participants through surveys and interviews, focusing on factors such as age, income, physical activity, healthcare access, and food availability. The findings revealed significant correlations between these factors and obesity rates, highlighting the importance of socioeconomic conditions in urban obesity. The study emphasizes the need for targeted interventions to address these contributing factors. These results provide valuable insights for developing effective obesity prevention and management strategies in urban areas. The advancement to knowledge in this study lies in identifying the socioeconomic factors and health management strategies that contribute to obesity in urban populations, providing insights for targeted interventions.

## Background:

Obesity is a growing public health issue worldwide, particularly in urban populations. It is characterized by excessive fat accumulation that presents a risk to health [1]. The prevalence of obesity has risen significantly in the last few decades, especially in cities where lifestyle changes, sedentary behavior and poor dietary habits are common [2]. Urbanization has brought about several changes that have directly contributed to the rise in obesity rates [3]. As cities expand, people's diets become more reliant on fast food and processed items that are high in calories and low in nutritional value [4]. Additionally, the fast-paced lifestyle in urban settings often leads to less physical activity, contributing to an increase in weight gain [5]. Socioeconomic factors play a crucial role in the prevalence of obesity in urban populations [6]. Individuals from lower socioeconomic backgrounds often face barriers to healthy living, such as limited access to fresh fruits and vegetables, affordable healthcare, and safe spaces for physical activity [7]. These factors contribute to unhealthy eating habits and sedentary lifestyles. Furthermore, economic stress can lead individuals to choose cheaper, calorie-dense foods that are less nutritious [8]. In contrast, wealthier individuals often have better access to healthier food options, healthcare, and fitness resources, which can help maintain a healthy weight [9]. The urban environment also shapes obesity-related behaviors through the built environment and available infrastructure. Cities with limited green spaces or walking areas may discourage outdoor activities, leading to less physical movement among residents [10]. Moreover, marketing and advertising of unhealthy foods in urban areas are often targeted at vulnerable populations, making it harder for people to make informed dietary choices. Cultural shifts and social norms in cities also affect eating patterns, with social gatherings often revolving around high-fat, high-sugar foods [11]. Therefore, it is of interest to determine the key factors contributing to obesity in urban populations and to explore strategies for effective health management in these settings.

## Methodology:

This study aimed to assess the health management strategies and socioeconomic factors contributing to obesity in urban populations. A combination of quantitative and qualitative research methods was employed to gather comprehensive data on this issue. A cross-sectional study design was used to collect data at a single point in time. This design enabled an in-depth assessment of factors contributing to obesity in urban populations and provided insights into current health management strategies in these areas. The target population for this study consisted of adult individuals (aged 18 and above) living in urban areas. A sample size of 150 participants was selected to ensure a manageable yet diverse representation of the population. This sample size was sufficient to gather meaningful data and provide insights into the socioeconomic factors influencing obesity. The participants were selected from diverse urban areas to ensure diversity in socioeconomic background, age, gender, and geographic location. A stratified random sampling method was used to ensure the sample accurately represented the urban population. The metropolitan areas were divided into different strata based on demographic factors such as income level, education, and employment status. Random sampling was then conducted within each stratum to select participants. This technique ensured that a wide range of socioeconomic backgrounds and health conditions were represented. Data were collected through surveys and in-depth interviews. The surveys were structured to gather information on participants' demographics, dietary habits, physical activity levels, and their awareness of obesity-related health risks. The questions also focused on participants' access to healthcare, the availability of healthy food, and local infrastructure, such as parks or gyms, that could influence physical activity. In addition to the surveys, in- depth interviews were conducted with a subset of 30 participants to explore their personal experiences with obesity and health management strategies. These interviews provided qualitative insights into the social, cultural, and psychological factors contributing to obesity. Participants were asked to evaluate their own health management strategies, including any weight management programs they had participated in, their knowledge of obesity prevention, and the effectiveness of health interventions available in their area. The quantitative data collected from the surveys were analyzed using descriptive and inferential statistics. Descriptive statistics summarized the demographic and socioeconomic characteristics of the participants, while inferential statistics, such as chi-square tests and logistic regression, were used to identify significant relationships between socioeconomic factors and obesity rates. The qualitative data from the interviews were analyzed thematically to identify common patterns and themes related to health management and the factors contributing to obesity.

## Results:

The results of this study are based on the analysis of various factors contributing to obesity in urban populations. The following tables present the data on age, income level, physical activity, healthcare access, and food access, along with the corresponding obesity rates. The obesity rates across different age groups are summarized in Table 1 (see PDF). The highest obesity rate was observed in the 56+ age group (58%), followed by the 46-55 age group at 55%. The obesity rate decreased as the age group got younger, with the lowest obesity rate found in the 18-25 age groups (35%). Table 2 (see PDF) shows the obesity rates based on income levels. Individuals in the middle- income group had the highest obesity rate (50%), followed by the low-income group at 40%. The high-income group had the lowest obesity rate, at 30%. Table 3 (see PDF) reveals a strong correlation between physical activity and obesity rates. The obesity rate was highest among individuals with low physical activity (60%), whereas it was lowest among those with high physical activity (20%). The relationship between healthcare access and obesity is shown in Table 4 (see PDF). Participants with poor healthcare access had an obesity rate of 50%, while those with excellent healthcare access had the lowest obesity rate (30%). Table 5 (see PDF) demonstrates the connection between food access and obesity rates. Those with limited access to healthy food had the highest obesity rate (55%), followed by individuals with adequate food access (40%). People with abundant access to healthy food had the lowest obesity rate (30%). The data indicate several key patterns related to obesity in urban populations:

## Age group and obesity: 

The obesity rate increased with age, with the highest rates observed in the 56+ age group. This trend suggests that older individuals in urban areas may face more challenges in maintaining a healthy weight due to factors such as decreased physical activity and changes in metabolism.

## Income level and obesity:

The middle-income group had the highest obesity rate, indicating that individuals in this income bracket may be more likely to experience obesity compared to those in lower or higher-income groups. This could be due to factors such as limited access to quality food or healthcare, and lifestyle habits tied to income constraints.

## Physical activity and obesity:

A clear inverse relationship was observed between physical activity and obesity. Those who engaged in low physical activity had the highest obesity rate (60%), while those who engaged in high physical activity had the lowest obesity rate (20%). This highlights the importance of promoting physical activity as a strategy to combat obesity.

## Healthcare access and obesity:

People with poor healthcare access had a significantly higher obesity rate (50%) than those with excellent healthcare access (30%). This suggests that access to healthcare may influence weight management and the ability to prevent or treat obesity.

## Food access and obesity:

Limited access to healthy food was strongly associated with higher obesity rates, with 55% of participants in this category being obese. In contrast, those with abundant access to nutritious food had the lowest obesity rate (30%). This underlines the importance of improving access to healthy food options in urban environments to prevent obesity.

## Discussion:

This study aimed to assess the health management strategies and socioeconomic factors contributing to obesity in urban populations. The findings revealed significant associations between age, income level, physical activity, healthcare access, and food availability with obesity rates. These results align with existing literature that highlights the complex interplay of socioeconomic determinants in urban obesity. Consistent with previous research, our study found that older age groups exhibited higher obesity rates. This trend is supported by studies such as Arambepola *et al.* (2008) [12], which observed that urban living is associated with obesity independently of other demographic, socioeconomic, and lifestyle characteristics. Additionally, our findings corroborate the work of Keetile *et al.* (2019) [13], who identified socioeconomic and behavioral determinants of obesity among adults in Botswana, emphasizing the role of education and physical activity. Income level emerged as a significant predictor of obesity, with middle-income individuals exhibiting higher obesity rates. This observation aligns with Titis *et al.* (2023) [14], who found that income was the main predictor of childhood obesity in urban areas of England. Their study highlighted the importance of addressing income disparities to mitigate obesity risks. The inverse relationship between physical activity and obesity observed in our study is consistent with findings by Lee *et al.* (2021) [15], who reported that urban adolescents with lower levels of physical activity had higher obesity rates. This underscores the need for urban planning that promotes opportunities for physical activity. Healthcare access was another critical factor, with poor access correlating with higher obesity rates. This is supported by Saxon *et al.* (2019) [16], who demonstrated that offering low-cost weight management tools in primary care led to significant weight loss among low-income patients. Their study emphasizes the importance of accessible healthcare services in obesity management. Finally, our study found that limited access to healthy food was strongly associated with higher obesity rates. This finding is in line with research by Stankov *et al.* (2025) [17], who discussed the role of food systems and diet in obesity, particularly in socioeconomically deprived urban neighborhoods. Improving food access is crucial in addressing urban obesity.

## Conclusion:

We show the significant roles that age, income level, physical activity, healthcare access, and food availability play in shaping obesity rates in urban populations. Addressing these factors through targeted health management strategies can help reduce obesity and its related health risks.
